# Integrated transcriptomic and metabolomic profiles analysis reveals a potential gene−metabolite network associated with anthocyanin−mediated color variation in maize kernels

**DOI:** 10.3389/fpls.2026.1828668

**Published:** 2026-05-13

**Authors:** Junhao Ran, Ruirui Hu, Xuanyu Liu, Ziyi Fu, Zhiqiang He, Guangtong Xing, Yi He, Dongpu Ji, Chaofeng Li, Xiupeng Mei, Lian Zhou

**Affiliations:** 1 Maize Research Institute, College of Agronomy and Biotechnology, Southwest University, Beibei, Chongqing, China; 2 Department of Plant Science, College of Agriculture, Missouri State University, Springfield, MO, United States

**Keywords:** anthocyanins, kernel, maize, metabolome, transcriptome

## Abstract

Anthocyanins are important flavonoid pigments responsible for coloration in maize kernels and are associated with nutritional and health-promoting properties. This study integrated metabolomic and transcriptomic analyses to investigate the biochemical and genetic basis of kernel pigmentation in three maize inbred lines with distinct kernel colors: yellow (Yellow−K), red (Red−K), and purple (Purple−K). Phenotypic and biochemical analyses revealed that anthocyanins accumulated exclusively in the pericarp of Red−K and in both pericarp and aleurone layers of Purple−K, with total anthocyanin content highest in Purple−K. Metabolomic profiling identified 1,845 differentially accumulated metabolites (DAMs) common across all comparisons, with flavonoids and anthocyanins significantly more abundant in colored kernels. Genotype-specific divergence in anthocyanin biosynthetic flux and decorative modifications showed that Purple−K specialized in malonylation and sambubiosylation, with massive accumulation of Pg3DiMalG; Red−K specialized in rutinosylation and 5−O−glycosylation. Transcriptome analysis identified differentially expressed genes (DEGs), of which 22 structural genes, including *PALs*, *FHT1*, *PR1*, *A1*, *A2*, *BZ1*, *BZ2*, were coordinately upregulated in Purple−K and Red−K, showing expression patterns highly correlated with metabolite levels. Integrated omics analysis further identified 101 transcriptional regulators, including 4 MYB and 10 bHLH transcription factors (e.g., *R1*, *PL1*) with expression correlated with anthocyanin accumulation. Downregulation of *JAZ* repressors in pigmented kernels, along with upregulation of *R1* and other bHLH factors, were observed. Together, these findings suggest that the differential accumulation of specific anthocyanin metabolites, coordinated upregulation of structural genes, and involvement of key transcription factors collectively associated with kernel color variation. This study provides insights into the potential molecular mechanisms underlying anthocyanin-based pigmentation in maize and provides a useful resource for breeding programs aiming to improve nutritional quality and visual traits in maize germplasm.

## 1 Introduction

Anthocyanins represent a diverse class of specialized metabolites with significant biological importance in plants. As the end products of flavonoid metabolism, they provide coloration to various plant organs, including flowers, leaves, fruits, and seeds. Anthocyanins contribute to critical physiological functions such as facilitating pollination and enhancing stress tolerance (Buer et al., 2010). Additionally, these compounds serve as natural, structurally diverse pigments that offer safer potential alternatives to commercial chemical synthetic dyes (Amchova et al., 2015). Moreover, anthocyanins are valued as natural dietary components and have been recognized for their beneficial effects on human health (De Nisi et al., 2021; Peniche-Pavía et al., 2022).

Most maize (*Zea mays* L.) cultivars cultivated worldwide typically exhibit green aerial parts and yellow to orange-yellow kernels, a coloration primarily attributed to the presence of carotenoids. However, certain cultivars display anthocyanin pigmentation across various tissues. Among these, kernel tissues have received particular attention for studying the diversity of anthocyanin production (Dooner et al., 1991). In maize kernel, anthocyanins predominantly accumulated in pericarp tissue and/or in the aleurone (Li et al., 2012; Harakotr et al., 2014a).

The structural and regulatory genes involved in anthocyanin biosynthesis have been identified and functionally described (Petroni et al., 2014). Generally, the pathway begins with phenylalanine and proceeds through a series of structural genes, including phenylalanine ammonia lyase (PAL), cinnamate 4-hydroxylase (C4H), 4-coumarate: coenzyme A ligase (4CL), chalcone synthase (CHS), chalcone isomerase (CHI), flavanone 3-hydroxylase (F3H), flavonoid 3’-hydroxylase (F3’H), flavonoid 3’5’-hydroxylase (F3’5’H), dihydroflavonol reductase (DFR), anthocyanidin synthase (ANS) (Castillejo et al., 2020; Luo et al., 2018; Liu et al., 2021). Following the biosynthesis of unstable anthocyanidins, glycosylation and acylation are critical downstream modifications that determine anthocyanin structural diversity, stability, and subcellular localization (Yonekura-Sakakibara and Hanada, 2011; Nakayama et al., 2003). Glycosylation is primarily catalyzed by UDP-glucose:flavonoid 3-O-glucosyltransferase belonging to the UGT family, which stabilizes anthocyanidins by adding a glucose moiety to the 3-hydroxyl position (Bowles et al., 2006). In maize, the *Bronze1* (*BZ1*) locus encodes UFGT; its mutation causes bronze kernels due to accumulation of unstable, unglycosylated anthocyanidins (Ralston et al., 1988; Zhao and Dixon, 2010; Fedoroff et al., 1984). Acylation, catalyzed by anthocyanin acyltransferases (AATs), attaches aromatic or aliphatic acyl groups to the sugar moieties, predominantly at the 6-O position of 3-O-glucose (D'Auria, 2006; Luo et al., 2007; Suzuki et al., 2004). This modification enhances anthocyanin stability, promotes blue-shifted color via intramolecular co-pigmentation, and facilitates vacuolar uptake (Fukuchi-Mizutani et al., 2003; Yoshida et al., 2009; Mueller and Walbot, 2001). In maize, *AAT1* is co-expressed with biosynthetic genes, and malonylation is the predominant acylation form, with di-malonylated pelargonidin identified as a major pigment in purple varieties (Paulsmeyer and Juvik, 2021). Finally, the trafficking and vacuolar sequestration of anthocyanins are primarily mediated by glutathione S-transferases (GSTs). In maize, the *Bronze2* (*BZ2*) locus encodes a GST facilitating the conjugation of reduced glutathione to anthocyanins such as cyanidin 3-glucoside, a necessary step for their vacuolar import (Marrs et al., 1995).

The structural genes involved in anthocyanin biosynthesis are regulated by several families of transcriptional factors (TFs) (Zhang et al., 2016). The process is primarily regulated by the MYB-bHLH-WD40 complex, which consists of three key protein components: MYB (V-myb myeloblastosis viral oncogene homolog), bHLH (Basic helix-loop-helix), and WD-repeat (Sharma et al., 2011). In maize, two anthocyanin-related MYB transcription factors, *C1* and *Pl*, play crucial roles in tissue-specific anthocyanin accumulation (Cone et al., 1993; Paz-Ares et al., 1987; Cone et al., 1986; Paz-Ares et al., 1986). Additionally, the bHLH regulatory factors *Booster1* (*B1*) and *intensifier1* (*in1*) are predominantly associated with anthocyanin regulation in plant tissues (Styles and Coe, 1986; Burr et al., 1996). Overexpression of the maize MYB and bHLH transcription factors *ZmC1* and *ZmR* in wheat has been shown to enhance anthocyanin content (Riaz et al., 2019). In *Arabidopsis*, overexpression of a maize WD40 family transcription factor *pale aleurone color 1* (*pac1*) functionally complements *ttg1* mutant, restoring the levels of both anthocyanins and proanthocyanins (Carey et al., 2004; Chatham and Juvik, 2021).

Although the anthocyanin biosynthetic pathways have been elucidated in many plant species, the molecular mechanisms governing its regulation in maize remain fragmented. Recent multi-omics studies have begun to reveal the regulatory networks underlying anthocyanin accumulation in maize and other cereal crops (Lu et al., 2023; Jiang et al., 2023; Wang et al., 2025; Zhang et al., 2025b). However, a systematic integration of metabolic profiles with global gene expression patterns across genetically distinct, colored maize inbred lines is lacking, which is crucial for identifying the key regulatory nodes and limiting steps in the pathway. This study performed an integrated metabolomic and transcriptomic comparison using kernels of three maize inbred lines with different colors: Yellow-K, Red-K, and Purple-K. Beyond identifying type of anthocyanins in the maize, we reveal structural genes and transcription factors co-expressed with anthocyanin accumulation, and potential associations with the distinct pigmentation patterns. The multi-omics study seeks to provide insights into regulatory mechanisms that may determine anthocyanin composition and concentration in maize kernels, providing a resource for fundamental research and potential metabolic engineering applications.

## 2 Materials and methods

### 2.1 Plant materials

Three stable maize inbred lines, designated Yellow−K (yellow kernel), Red−K (red kernel), and Purple−K (purple kernel), were originally derived among local landraces preserved in rural areas of southwest area, China. All lines were grown under standard irrigation and fertilization regimes at the Southwest University experimental farm. Kernels were harvested at 25 DAP (days after pollination) and used for subsequential analyses.

### 2.2 Quantification of anthocyanin content

Anthocyanins were extracted and quantified from maize kernels following a previously described protocol (Riaz et al., 2019), with detailed procedures outlined below. Fresh kernels (0.100 g) were ground into a 1 mL extraction solution [Methanol: HCl (99.9:0.1)]. The samples were vortexed for 30 s and centrifuged at 4 °C and 12,000 g for 2 min. The pellet was rinsed twice with extraction solution and the supernatant liquid volume was brought to a final volume of 5 mL. Absorbance was measured at 530 nm and 600 nm using a spectrophotometer (Millipore, MA, USA). The relative anthocyanin content was calculated according to the formula: Q = V × (A_530_-A_600_)/M. where Q represents anthocyanin content (units g^-^¹ fresh weight), V is the final volume (mL), A_530_ and A_600_ are the absorbance values at the respective wavelengths, and M is the sample fresh weight (g). The extraction solution was used as a blank control for all measurements.

### 2.3 Metabolite identification and quantification

Samples were harvested at 25 days after pollination (DAP) from three maize cultivars: Yellow−K, Red−K, and Purple−K, each with three biological replicates. All subsequent steps of sample processing, metabolite extraction, and LC−MS analysis were conducted by Beijing BioMarker Technologies Corporation (Beijing, China) following their standardized procedures. Metabolite profiling was performed on an Acquity I-Class PLUS ultra-performance liquid chromatography (UPLC) coupled with Waters Xevo G2-XS Q-Tof mass spectrometer. Raw data were processed using Progenesis QI software (Waters Corporation). Normalization was performed using total peak area per sample. Quality control (QC) samples, prepared by pooling aliquots of all samples, were analyzed at regular intervals; the coefficient of variation (CV) for metabolite intensities in QC samples was less than 20% for most detected features. After metabolite identification, orthogonal partial least squares-discriminant analysis (OPLS-DA) was performed to distinguish metabolic profiles among sample groups. Differentially accumulated metabolites (DAMs) were defined as those meeting the following criteria: log_2_FC ≥ 1, P-value < 0.05, and variable importance in projection (VIP) score ≥ 1. Enrichment analysis of the DAMs was subsequently conducted based on the Kyoto Encyclopedia of Genes and Genomes (KEGG) database, with significance assessed using a hypergeometric distribution test.

### 2.4 Transcriptome analysis

Kernel samples were collected at 25 DAP from three maize kernel cultivars (Yellow-K, Red-K, and Purple-K). Three biological replicates were used for all samples. The RNA sequencing was performed by Beijing BioMarker Technologies Corporation (Beijing, China). The cDNA library was sequenced on an Illumina NovaSeq platform (HiSeqTM 2500). Raw sequencing data were processed on the BMKCloud online platform (www.biocloud.net) to obtain clean reads. Clean reads were aligned to the maize reference genome (Zm_B73_REFERENCE_NAM_5.0 from maizeGDB) via HISAT2 system. Gene expression levels were quantified using FPKM (fragments per kilobase of transcript per million fragments mapped). Differentially expressed genes (DEGs) were identified with DESeq2 based on the criteria of Fold Change ≥2 and adjusted P-value < 0.01, with Benjamini and Hochberg’s approach correction to control the false discovery rate (FDR).

### 2.5 Comprehensive analysis of the transcriptome and metabolome

To elucidate the relationship between gene expression and metabolite accumulation and to predict the molecular mechanism of maize kernel coloration, key DEGs and DAMs were filtered by their functional annotations and association with relevant biosynthesis pathways (phenylpropanoid, flavonoid, anthocyanin, isoflavonoid, flavone and flavonol biosynthesis; KEGG pathways Ko00940–Ko00944) and through correlation analysis. The selected DEGs and DAMs were then jointly mapped to KEGG pathways to reconstruct the anthocyanin biosynthesis network in maize kernels. WGCNA was performed using the WGCNA R package following established protocols. Pearson correlation coefficients were calculated for all pairwise comparisons, and the correlation matrix was transformed into an adjacency matrix using a power function (soft−thresholding power β = 8, scale−free R² > 0.85). Modules were identified using average linkage hierarchical clustering based on topological overlap dissimilarity, with a dynamic tree−cutting algorithm. Module eigengenes were calculated as the first principal component of each module. Correlations between gene and metabolite modules were calculated using Pearson correlation; modules with |r| > 0.5 and P < 0.01 were considered significantly correlated.

### 2.6 RNA extraction and quantitative real-time PCR

Total RNA was isolated from leaf samples using the RNAprep Pure Plant Kit (Tiangen, Beijing, China) following the manufacturer’s protocol. cDNA synthesis was performed with the RevertAid First Strand cDNA Synthesis Kit (Thermo Scientific, USA). Quantitative real−time PCR (qRT−PCR) assays were conducted on a CFX96 Touch™ Cycler system (Bio−Rad, USA) with SYBR Premix Ex Taq™ II (Takara, China). Gene expression levels were normalized and calculated using the 2^−ΔΔCt^ method. *ZmACTIN3* and *ZmGAPDH* were used as the internal reference genes. Primer sequences are provided in **Supplementary Table 1**. Statistical significance was determined using Student’s t-test (* *P* < 0.05, ** *P* < 0.01). Error bars represent standard deviation (SD) of three biological replicates.

## 3 Results

### 3.1 Morphology observation and quantification of total anthocyanin content in maize kernels

**Figure 1:**
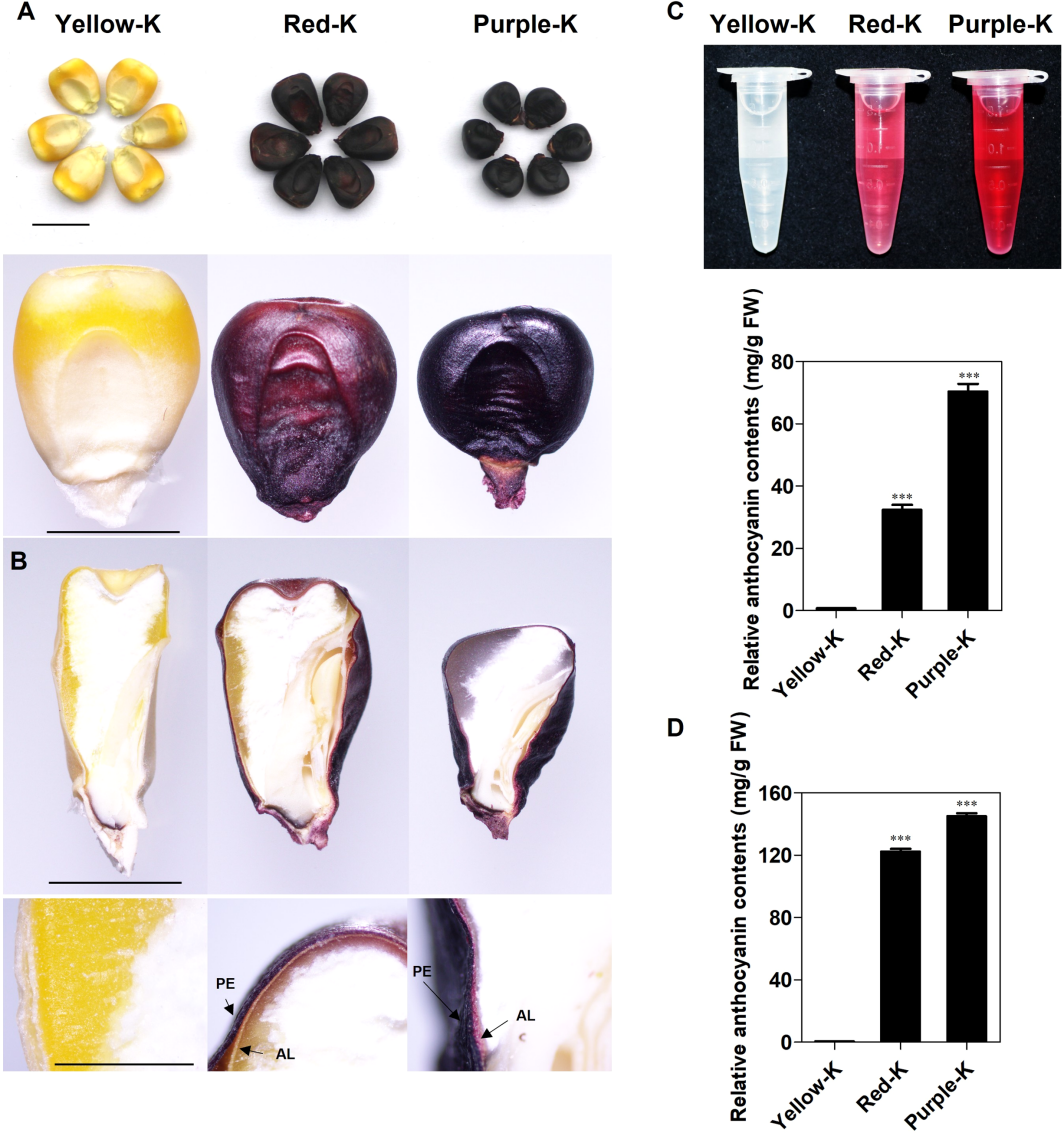
Phenotype and relative anthocyanin content of three varieties of different color maize kernels. **(A)** yellow (Yellow-K), red (Red-K) and purple (Purple-K) kernels from left to right **(B)** Microscopic observation of longitudinally sectioned kernels. PE, pericarp; AL, aleurone. Scale bar = 1 cm. **(C)** Relative anthocyanin content in different color maize kernels, respectively. **(D)** Relative anthocyanin content of pericarp and aleurone layers in different color maize kernels, respectively. Values are means ± SD (n=3). ***P < 0.001 indicates significant difference compared to Yellow-K.

To characterize the morphology features and anthocyanin content of maize kernels, three inbred lines with different kernel color, Yellow-K (yellow kernel), Red-K (red kernel), and Purple-K (purple kernel) were selected for this study (**Figure 1A**). To determine the distribution of anthocyanin-pigmented tissues, mature kernels were longitudinally sectioned and examined. No anthocyanin was detected in the pericarp or aleurone layers of Yellow-K. In contrast, anthocyanins accumulated exclusively in the pericarp layer of Red-K, whereas both the pericarp and aleurone layers exhibited anthocyanin accumulation in Purple-K (**Figure 1B**).

To compare anthocyanin levels among the different kernel types, total anthocyanin contents were quantified. Visual assessment indicated that Purple-K and Red-K kernels contained visibly more pigment than Yellow-K kernels (**Figure 1C**). Quantitative analysis revealed that Purple-K kernels possessed the highest anthocyanin content, approximately 70 mg/g in whole kernels and 140 mg/g in isolated pericarp and aleurone tissues. Red-K kernels exhibited lower anthocyanin levels, about 30 mg/g in whole kernels and 120 mg/g in the corresponding tissue fractions (**Figures 1C, D**). These results suggest that the observed differences in anthocyanin accumulation are attributable to germplasm-specific traits.

### 3.2 Metabolite analysis in differently colored maize kernels

**Figure 2:**
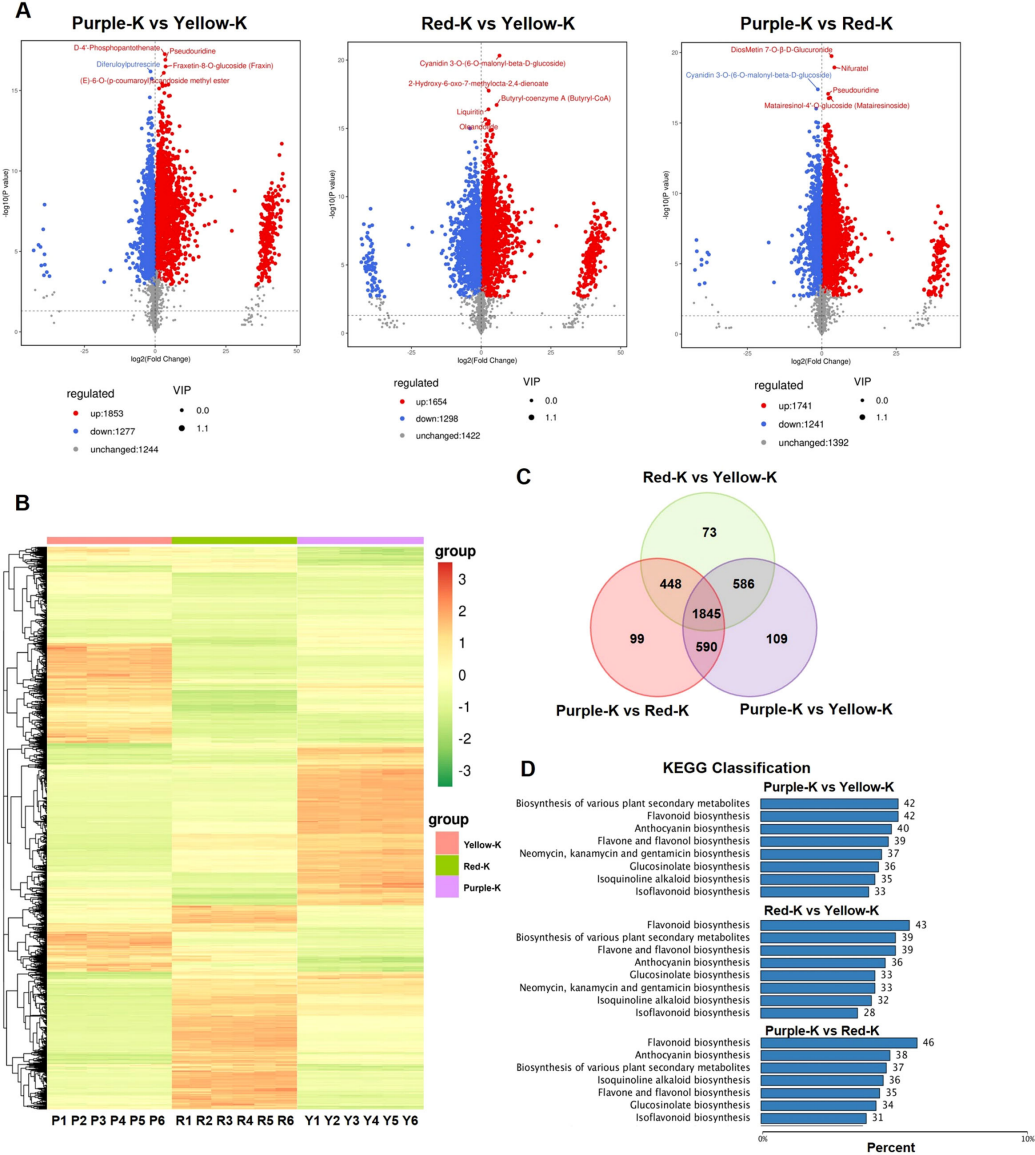
Differentially accumulated metabolites (DAMs) in three maize kernels and Kyoto Encyclopedia of Genes and Genomes (KEGG) pathway analysis. **(A)** Volcano plot of DAMs. Red and blue dots represent up- and downregulated metabolites, respectively. **(B)** Hierarchical clustering of 1845 annotated metabolites. The heatmap displays the relative abundance of metabolites across three kernel varieties. Colors represent log10-transformed normalized abundance values (from low to high, represented by a green-to-orange gradient). **(C)** Venn diagram depicts differentially accumulated metabolites between the three groups of maize kernel samples. **(D)** KEGG classification analysis.

To characterize the differential accumulation of anthocyanin species among differently colored maize kernels, a high-capacity metabolite library was established using LC-MS. In the pairwise comparisons, we identified 3,130 differentially accumulated metabolites (DAMs; 1,853 upregulated, 1,277 downregulated) in Purple−K versus Yellow−K, 2,952 DAMs (1,654 upregulated, 1,298 downregulated) in Red−K versus Yellow−K, and 2,982 DAMs (1,741 upregulated, 1,241 downregulated) in Purple−K versus Red−K, respectively (**Figure 2A**). Hierarchical clustering analysis (HCA) of samples and metabolites revealed distinct metabolic profiles in Purple-K and Red-K compared to Yellow-K, with group-specific metabolite accumulation patterns identified (**Figure 2B**). Venn diagram analysis indicated that 1,845 DAMs were common across all comparisons, while 109, 73, and 99 DAMs were uniquely identified in Purple-K vs. Yellow-K, Red-K vs. Yellow-K, and Purple-K vs. Red-K, respectively. Additionally, 586 DAMs were shared between Purple-K vs. Yellow-K and Red-K vs. Yellow-K comparisons (**Figure 2C**). KEGG enrichment analysis further highlighted significant differences in pathways related to secondary metabolism, including flavonoid, anthocyanin, flavone and flavonol, and isoflavone biosynthesis (**Figure 2D**). A total of 202 flavonoid-related metabolites were identified across the yellow, red, and purple kernel samples, categorized into nine classes: flavonoids (42), anthocyanins (30), flavones and flavonols (39), isoflavones (33), tannins (5), dihydroflavones (9), dihydroflavonols (5), chalcones (18), flavonoid carbonoside (3), and proanthocyanidins (2), and flavanols (16). Among these, flavonoids and anthocyanins were found to be significantly more abundant in Purple-K compared to Red-K and Yellow-K (**Supplementary Table 2**).

### 3.3 Comparative analysis of transcriptome profiles among maize kernel samples

**Figure 3:**
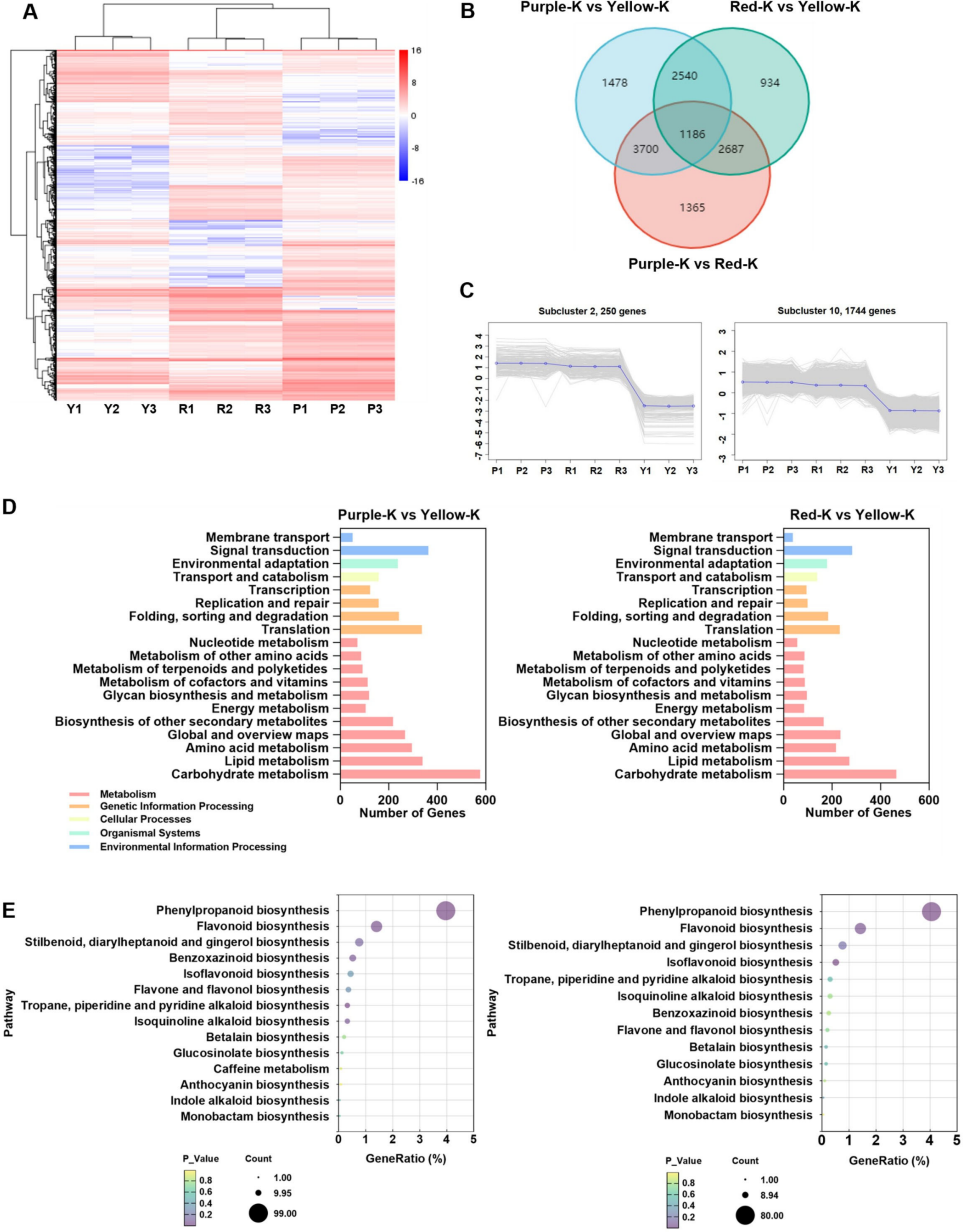
Differentially expressed genes (DEGs) in three maize kernels and Kyoto Encyclopedia of Genes and Genomes (KEGG) pathway analysis. **(A)** Hierarchical clustering analysis of differentially expressed genes (DEGs). The heatmap displays the expression profiles of DEGs across three kernel varieties. Colors represent log10-transformed normalized abundance values (from low to high, represented by a blue-to-red gradient). **(B)** Venn diagram showing the number of DEGs between the three groups of maize kernel samples. **(C)** K-means clustering analysis of DEGs according to the expression profiles. **(D)** KEGG classification analysis. **(E)** KEGG enrichment analysis of biosynthesis of other secondary metabolites pathway.

To further investigate gene expression variations, transcriptomic sequencing was performed on the three maize kernel types. Comparative analysis revealed significant differentially expressed genes (DEGs) in the following pairwise comparisons: Purple-K vs. Yellow-K (8,904 DEGs; 5,173 upregulated, 3,731 downregulated), Red-K vs. Yellow-K (7,347 DEGs; 4,353 upregulated, 2,994 downregulated), and Purple-K vs. Red-K (12,767 DEGs; 6,427 upregulated, 6,340 downregulated) (**Supplementary Figure 1A**). Hierarchical clustering showed distinct transcriptomic profiles in Purple-K and Red-K compared to Yellow-K, with group-specific DEGs identified (**Figure 3A**). Venn diagram analysis indicated that 1,186 DEGs were common to all three comparisons, while 1,478, 934 and 1,365 DEGs were uniquely identified in Purple-K vs. Yellow-K, Red-K vs. Yellow-K, and Purple-K vs. Red-K comparisons, respectively. Additionally, 2,540 DEGs were shared between Purple-K vs. Yellow-K and Red-K vs. Yellow-K comparisons (**Figure 3B**). K-means clustering of DEGs categorized the genes into eleven clusters based on expression patterns. Four major kinetic clusters of co-expressed genes were identified and classified as follows: Cluster 2 & 10: upregulated in Purple-K and Red-K, downregulated in Yellow-K; Cluster 5: downregulated in Purple-K and Red-K, upregulated in Yellow-K; Cluster 7 & 9: upregulated in Purple-K, downregulated in Red-K and Yellow-K; Cluster 1 & 4: downregulated in Purple-K, upregulated in Red-K and Yellow-K (**Supplementary Figure 1B**). Notably, Clusters 2 and 10 included multiple genes associated with flavonoid and anthocyanin biosynthesis (**Figure 3C**). KEGG classification analysis was used to identify the associations of the DEGs with specific anthocyanin-related processes to further explore the molecular mechanism underlying metabolic changes in the anthocyanins. As shown in **Figure 3D**, biosynthesis of other secondary metabolites pathway was prominently enriched in both Purple-K vs. Yellow-K and Red-K vs. Yellow-K comparisons. In the Purple-K vs. Yellow-K group, 217 DEGs were annotated to this category, with 151 DEGs further assigned to anthocyanin-related pathways, including phenylpropanoid biosynthesis (99), flavonoid biosynthesis (35), isoflavone biosynthesis (8), flavone and flavonol biosynthesis (8), and anthocyanin biosynthesis (1). Similarly, in the Red-K vs. Yellow-K comparison, 165 DEGs were associated with biosynthesis of other secondary metabolites pathway, among which 122 DEGs were enriched in anthocyanin-related pathways, comprising phenylpropanoid biosynthesis (80), flavonoid biosynthesis (28), isoflavone biosynthesis (10), flavone and flavonol biosynthesis (3), and anthocyanin biosynthesis (1) (**Figures 3D, E**; **Supplementary Table 3**). These transcriptional differences might be closely associated with the distinct biosynthetic and phenotypic characteristics of the three maize kernel types.

### 3.4 Combined transcriptome and metabolome analysis of anthocyanin biosynthetic pathway

**Figure 4:**
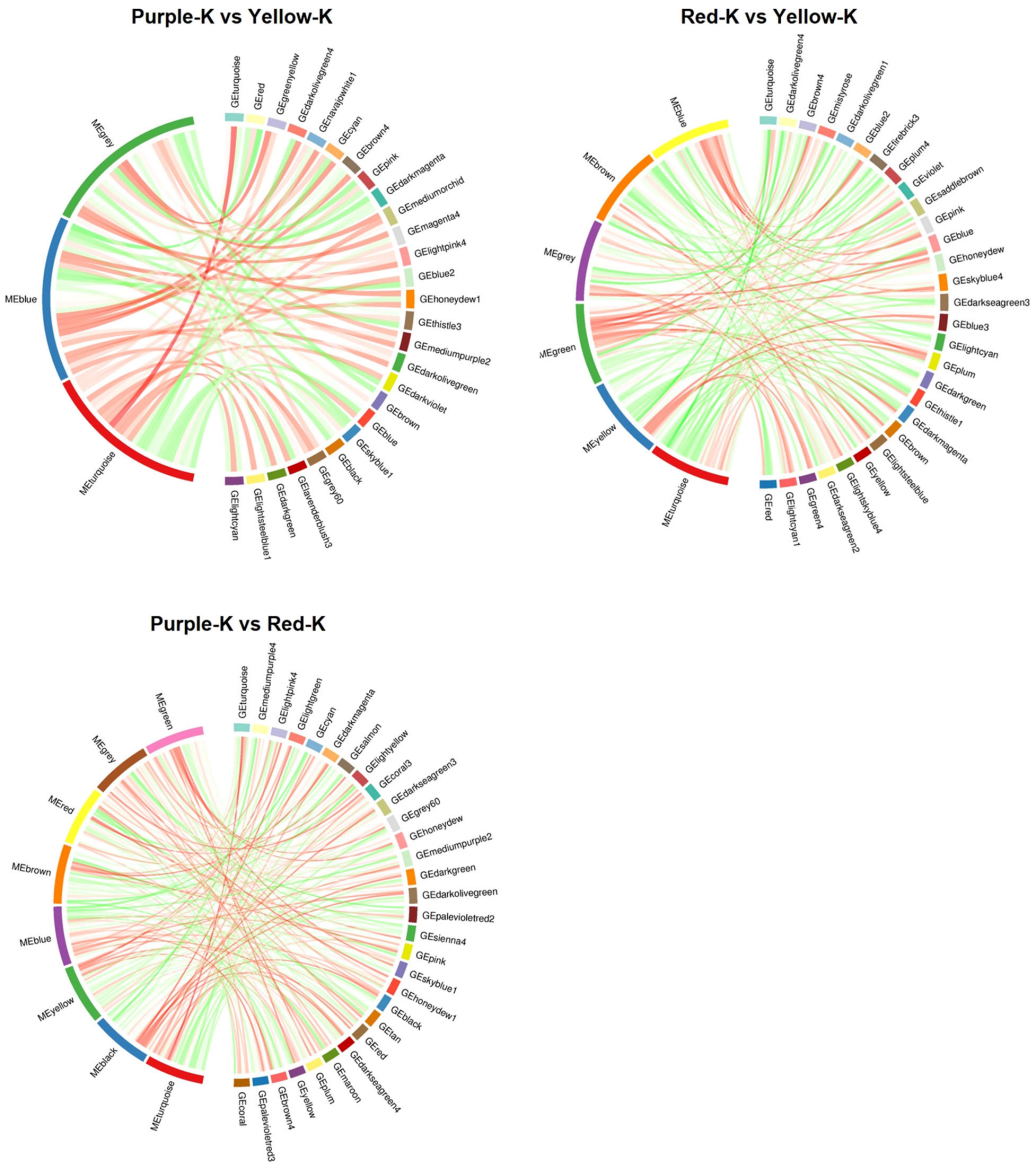
Correlation chord diagram of metabolite modules and gene modules between the three groups of maize kernel samples. The left and right semicircles represent metabolite modules and gene modules, respectively. Ribbon width indicates correlation strength (Pearson correlation coefficient), and ribbon color denotes the direction of correlation (red, positive; green, negative).

To clarify the metabolite and gene module correlation in maize kernels, a weighted gene co−expression network analysis (WGCNA) was conducted using filtered DEGs and DAMs, and visualized in a metabolite–gene module correlation chord diagram. In the comparisons of Purple−K vs. Yellow−K, Red−K vs. Yellow−K, and Purple−K vs. Red−K, the DAMs were clustered into 3, 6, and 8 metabolite modules, respectively. Each metabolite module showed high correlation with a corresponding gene module, indicated by distinct colors (**Figure 4**; **Supplementary Table 4**). In both Purple−K vs. Yellow−K and Purple−K vs. Red−K comparisons, the MEturquoise metabolite module exhibited a notably strong positive correlation (correlation coefficient > 0.5, CCP < 0.01) with the GEturquoise gene module. Conversely, in the Red−K vs. Yellow−K comparison, the MEturquoise module displayed a significant negative correlation (correlation coefficient > 0.5, CCP < 0.01) with the GEturquoise module. The MEturquoise module contained the highest number of metabolites associated with anthocyanins, flavonoids, flavones and flavonols, isoflavones, tannins, flavanols, dihydroflavones, dihydroflavonols, carboglycoside flavonoids, chalcones, and proanthocyanidins (**Supplementary Table 5**). These results suggest that genes within the MEturquoise module may be associated with anthocyanin biosynthesis; therefore, this module was selected for further investigation.

**Figure 5:**
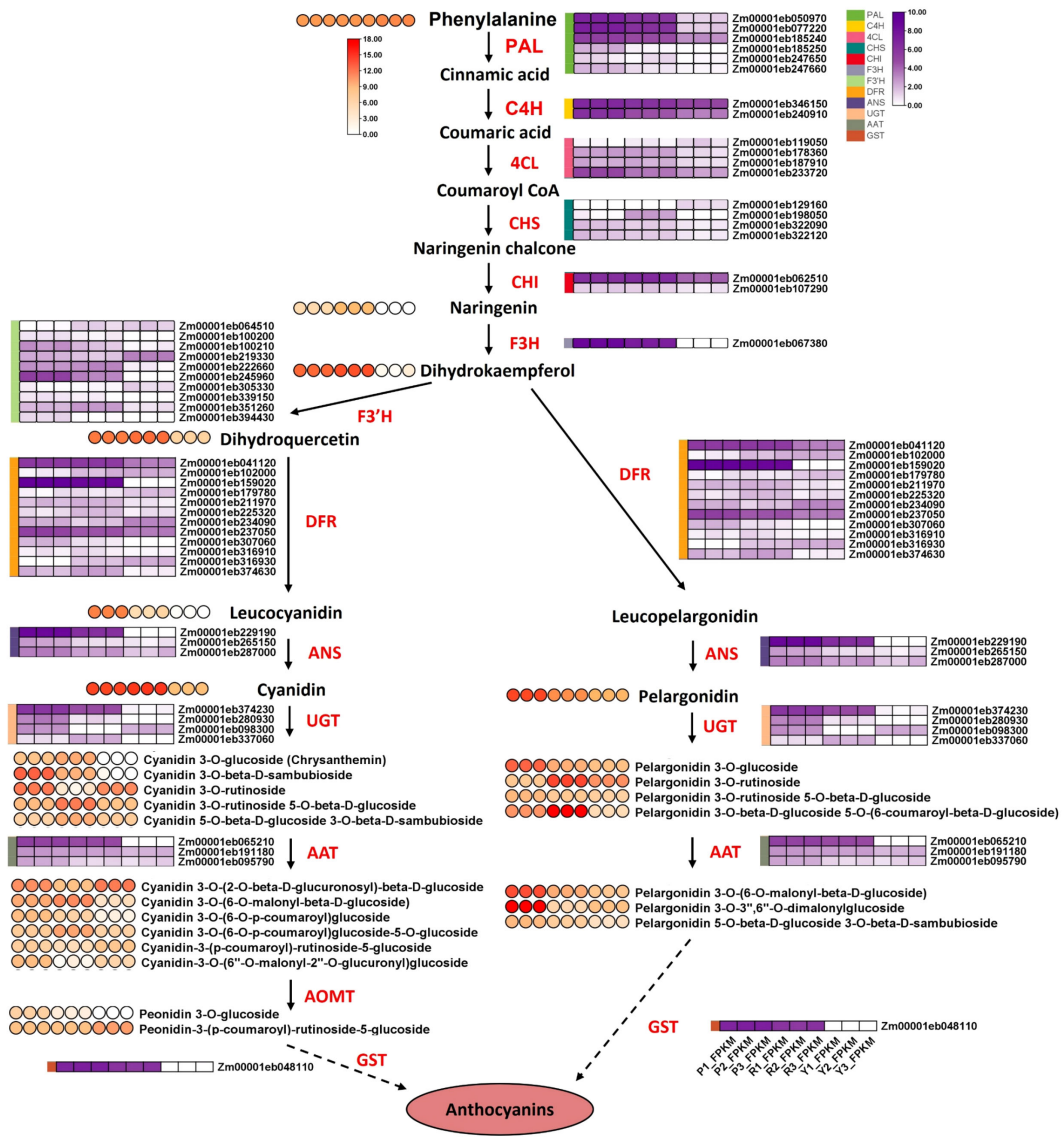
Genes and metabolites involved in the coloration anthocyanin pathway in three maize kernels. Values were normalized by converting to log10. Columns represent sample libraries and rows represent genes or metabolites. Rectangles represent genes of expression levels indicated by a white-to-purple gradient; circles represent metabolites with accumulation levels indicated by a white-to-red gradient. Enzymes in this pathway are shown as follows: PAL, phenylalanine ammonia lyase; 4CL, 4-coumarate-CoA ligase; CHS, chalcone synthase; CHI, chalcone isomerase; F3H, flavanone 3-hydroxylase; F3’H, flavonoid 3-hydroxylase; DFR, dihydroflavonol 4-reductase; ANS, anthocyanidin synthase; UGT, UDP-glucosyltransferase; GST, glutathione transferase; AOMT, flavonoid 3′,5′- methyltransferase; AAT, anthocyanin acyltransferases.

To examine the relationship between gene expression and metabolite accumulation and to investigate the color formation process in maize kernels, the anthocyanin biosynthesis pathway was reconstructed through integrated analysis of transcriptomic and metabolomic data (**Figure 5**). Based on combined omics profiles, a complete anthocyanin biosynthetic network was established, in which 52 structural genes were annotated and found to be differentially expressed among the three kernel types. These genes include *PAL* (6), *C4H* (2), *4CL* (4), *CHS* (4), *CHI* (2), *F3H* (1), *F3’H* (10), *DFR* (12), *ANS* (3), *UGT* (4), *AAT* (3) and *GST* (1) (**Figure 5**, **Supplementary Table 6**). Among them, 22 genes showed significant upregulation in both Purple−K and Red−K compared to Yellow−K and displayed coordinated expression with anthocyanin accumulation. These include 3 *PAL* genes (Zm00001eb050970 *PAL11*, Zm00001eb077220 *PAL6*, Zm00001eb185240 *PAL3*), 2 *C4H* genes (Zm00001eb346150 *CYP47*-*Cytochrome P450 47*, Zm00001eb240910), 2 *4CL* genes (Zm00001eb187910, Zm00001eb233720 *BM5*-*Brown midrib5*), 2 *CHS* genes (Zm00001eb322090 *CHLS3*-*Chalcone synthase3*, Zm00001eb322120), 1 *F3H* gene (Zm00001eb067380 *FHT1*-*Flavanone 3-hydroxylase 1*), 2 *F3’H* genes (Zm00001eb100210 *CYP12*, Zm00001eb245960 *PR1*-*Red aleurone1*), 2 *DFR* genes (Zm00001eb159020 *A1*-*Anthocyaninless1*, Zm00001eb237050), 2 *ANS* genes (Zm00001eb229190 *A2*-*Anthocyaninless2*, Zm00001eb287000 *ZAR8*-*Zea mays ARGOS8*), 2 *UGT* genes (Zm00001eb374230 *BZ1*, Zm00001eb280930 *UGT1*), 3 *AAT* genes (Zm00001eb065210 *AAT1*, Zm00001eb191180, Zm00001eb095790) and 1 *GST* gene (Zm00001eb048110 *BZ2*). Notably, *FHT1*, *PR1*, *A1*, *A2*, *BZ1*, *AAT1* and *Bz2* were nearly undetectable in Yellow−K, and *A2* expression in Purple−K was fivefold higher than in Red−K. Moreover, 1 *F3’H* gene (Zm00001eb394430 *FNS1*-*Flavone synthase1*), 1 *DFR* gene (Zm00001eb307060 *CNCR2*-*Cinnamoyl CoA reductase2*), 1 *ANS* gene (Zm00001eb265150 *FHT13*) were exclusively upregulated in Purple−K.

Targeted metabolomic analysis of three maize genotypes was performed to profile striking genotype-specific divergence in anthocyanin biosynthetic flux and decorative modifications (**Figure 5**; **Supplementary Table 7**). Yellow−K showed near-complete loss of naringenin, with L-phenylalanine accumulation (~1.8-fold higher than Purple−K/Red−K), suggesting a severe metabolic lesion at or upstream of CHS/CHI/F3H. Leucocyanidin was detected at levels >100-fold higher in Purple−K than Red−K/Yellow−K. For the pelargonidin branch, remarkable malonylation capacity was observed in Purple−K. Pelargonidin and its 3-O-glucoside (Pg3G) were ~10-fold higher in Purple−K than Red−K. Notably, pelargonidin 3-O-(6-O-malonyl-beta-D-glucoside) (Pg3MG) was >18-fold higher in Purple−K (~15,100 vs. ~820 in Red−K). Strikingly, pelargonidin 3-O-3’’,6’’-O-dimalonylglucoside (Pg3DiMalG) was >3,000-fold higher in Purple−K (~198,000 vs. ~57 in Red−K)—emerging as the single most abundant anthocyanin in Purple−K. This highly modified pelargonidin derivative could potentially contribute to the purple coloration through enhanced intermolecular stacking and stability. Cyanidin was abundant in both pigmented genotypes but was at higher levels in Red−K (~26,600 vs. ~16,800 in Purple−K). However, while Red−K accumulates primarily unmodified cyanidin, Purple−K channels cyanidin into diverse modified derivatives. Specifically, cyanidin 3-O-(6-O-p-coumaroyl)glucoside and cyanidin-3-O-(6’’-O-malonyl-2’’-O-glucuronyl)glucoside were >5-fold and >100-fold higher in Purple−K than Red−K, respectively, suggesting Purple−K-specific coumaroyltransferase and acyltransferase activities. Peonidin 3-O-glucoside was detected only in Purple−K (95–100) and virtually absent in Red−K (5–6) and Yellow−K (0), implying that anthocyanin O-methyltransferase (AOMT) activity may be present predominantly in Purple-K.

The accumulation patterns of these metabolites were generally consistent with the expression profiles of the associated structural genes. Together, these integrated omics data suggest that combinatorial expression of biosynthetic and modification genes may contribute to the distinct anthocyanin profiles and kernel coloration phenotypes in maize.

### 3.5 Analysis of potential transcription factors for regulating anthocyanin biosynthesis

**Figure 6:**
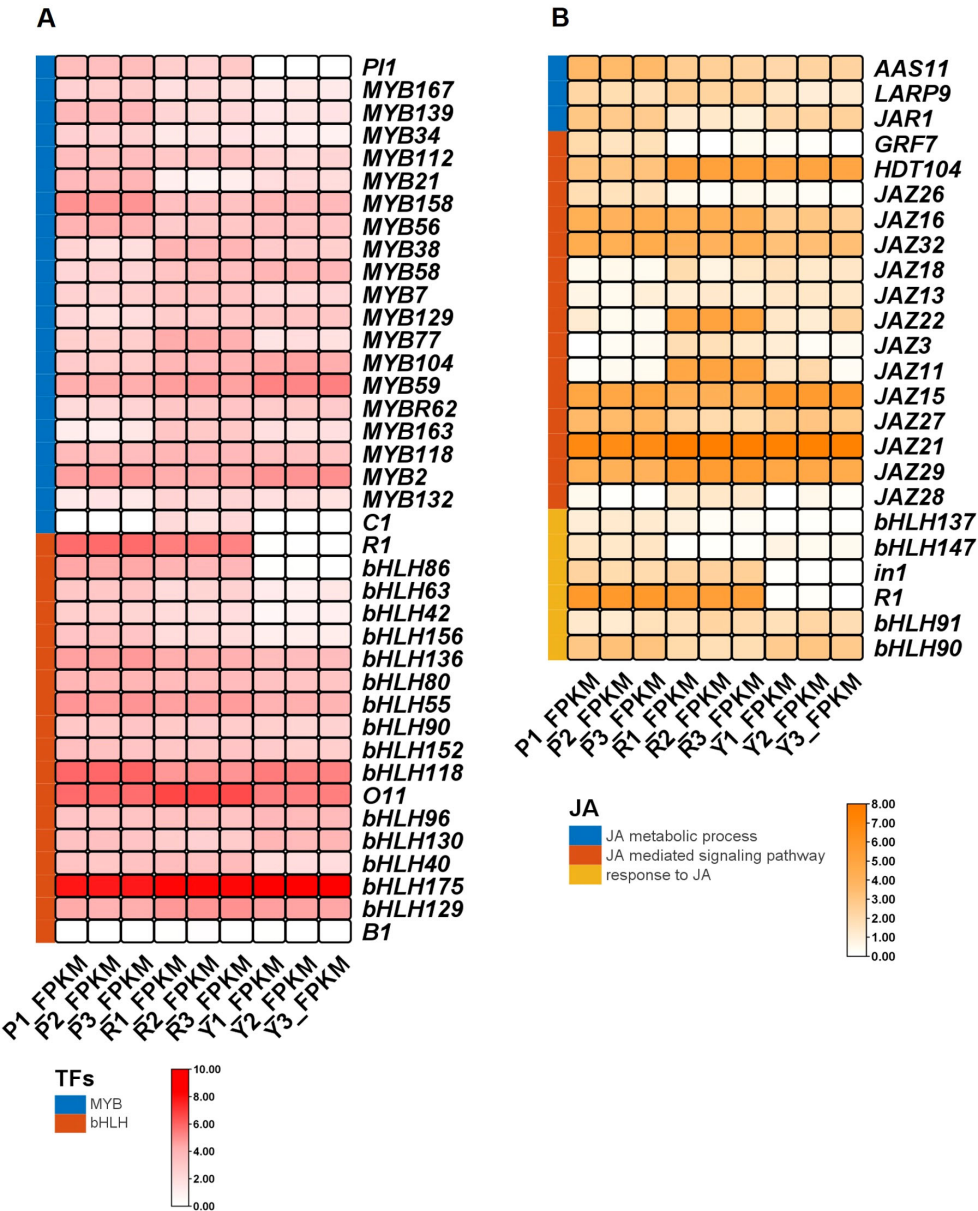
Heatmap showing differentially expressed **(A)** bHLH and MYB genes and **(B)** associated with the jasmonic acid biosynthetic process, metabolic process, mediated signaling pathway, and response in three maize kernels. Values were normalized by converting to log10. Low to high expression is indicated by color changes (white to red bHLH and MYB genes; white to orange JA related genes).

Previous studies have established that MYB and bHLH transcription factors play key roles in anthocyanin biosynthesis (Luo et al., 2018). Among the 6,440 DEGs identified in the GEturquoise module across all three comparisons, we identified 101 transcriptional regulators, including members of the AP2/ERF (21), MYB (20), bHLH (17), WRKY (8), NAC (14), and bZIP (21) families (**Supplementary Table 8**). Focusing on the two key TF families, we analyzed the expression profiles of MYB and bHLH genes potentially involved in anthocyanin regulation (**Figure 6A**; **Supplementary Table 9**). Co-expression analysis was performed using Pearson correlation between transcription factors and 22 core anthocyanin structural genes across nine samples (three biological replicates per genotype). Transcription factors with |r| > 0.8 and P < 0.01 were considered significantly co-expressed with the anthocyanin biosynthetic pathway. Based on these stringent criteria, we identified 14 transcription factors, including 4 MYB and 10 bHLH family members, that showed strong co-expression with anthocyanin structural genes (**Figure 6A**; **Supplementary Table 9**). The MYB factors included the known pericarp regulator *PL1* (Zm00001eb278680, r = 0.94) and three uncharacterized MYB genes: *MYB167* (Zm00001eb040160), *MYB139* (Zm00001eb095690), and *MYB34* (Zm00001eb147710) with correlation coefficients ranging from 0.89 to 0.93. *PL1* (Zm00001eb278680), a MYB regulator of pericarp pigmentation, was highly expressed in Purple-K, moderately expressed in Red-K, and undetectable in Yellow-K, consistent with its proposed role in pericarp anthocyanin biosynthesis. The bHLH factors included the well-characterized *R1* (Zm00001eb429330, r = 0.96) and nine additional bHLH genes (*bHLH86*, *bHLH63*, *bHLH42*, *bHLH156*, *bHLH136*, *bHLH80*, *bHLH55*, *bHLH90*, *bHLH152*) with correlation coefficients ranging from 0.81 to 0.94 (**Supplementary Table 9**). The bHLH factor *R1* (Zm00001eb429330) showed a similar pattern, with high expression in Purple-K and Red-K, but near-absence in Yellow-K (>300-fold reduction). Moreover, 3 MYB genes, *MYB21* (Zm00001eb266370), *MYB158* (Zm00001eb275490), and *MYB56* (Zm00001eb028820), and 1 bHLH gene, *bHLH118* (Zm00001eb289490) displayed preferential expression in Purple-K, with reduced expression in Red-K and Yellow-K (**Figure 6A**; **Supplementary Table 9**). These may be candidates for further investigation regarding their possible roles in an alternative regulatory complex associated with aleurone pigmentation.

### 3.6 Analysis of jasmonate signaling in differently colored maize kernels

To investigate the potential involvement of phytohormone signaling in regulating kernel pigmentation, we examined the expression of genes associated with the jasmonate (JA) pathway, a key regulator of anthocyanin biosynthesis in plants. Transcriptomic analysis revealed distinct pattern of JA-related gene expression changes in three kernels. Several JAZ repressor genes, which function as negative regulators of JA signaling, showed differential expression across genotypes. 2 JAZ repressor genes, *ZmJAZ18* (Zm00001eb048780) exhibited approximately 4-fold lower expression in Purple-K compared to Yellow-K, while *ZmJAZ13* (Zm00001eb100130) showed modestly reduced expression in both pigmented genotypes relative to Yellow-K (**Figure 6B**; **Supplementary Table 10**). However, *ZmJAZ22* (Zm00001eb009620), *ZmJAZ11* (Zm00001eb168150), *ZmJAZ21* (Zm00001eb048740) and *ZmJAZ29* (Zm00001eb223580) showed reduced transcript levels in Purple-K compared to Red-K but not to Yellow-K (**Figure 6B**; **Supplementary Table 10**). Four *bHLH* genes known to be targets of *JAZ* repression showed significantly elevated expression in pigmented kernels. *R1* (Zm00001eb429330) and *in1* (Zm00001eb303250) showed higher expression in both Purple-K and Red-K. *bHLH137* (Zm00001eb055830) and *bHLH147* (Zm00001eb298040) showed higher expression only in Purple-K relative to Yellow-K (**Figure 6B**; **Supplementary Table 10**). The coordinated upregulation of these bHLH factors in pigmented kernels suggests their potential involvement in JA-mediated anthocyanin biosynthesis.

### 3.7 Verification of RNA-Seq results by qRT-PCR

**Figure 7:**
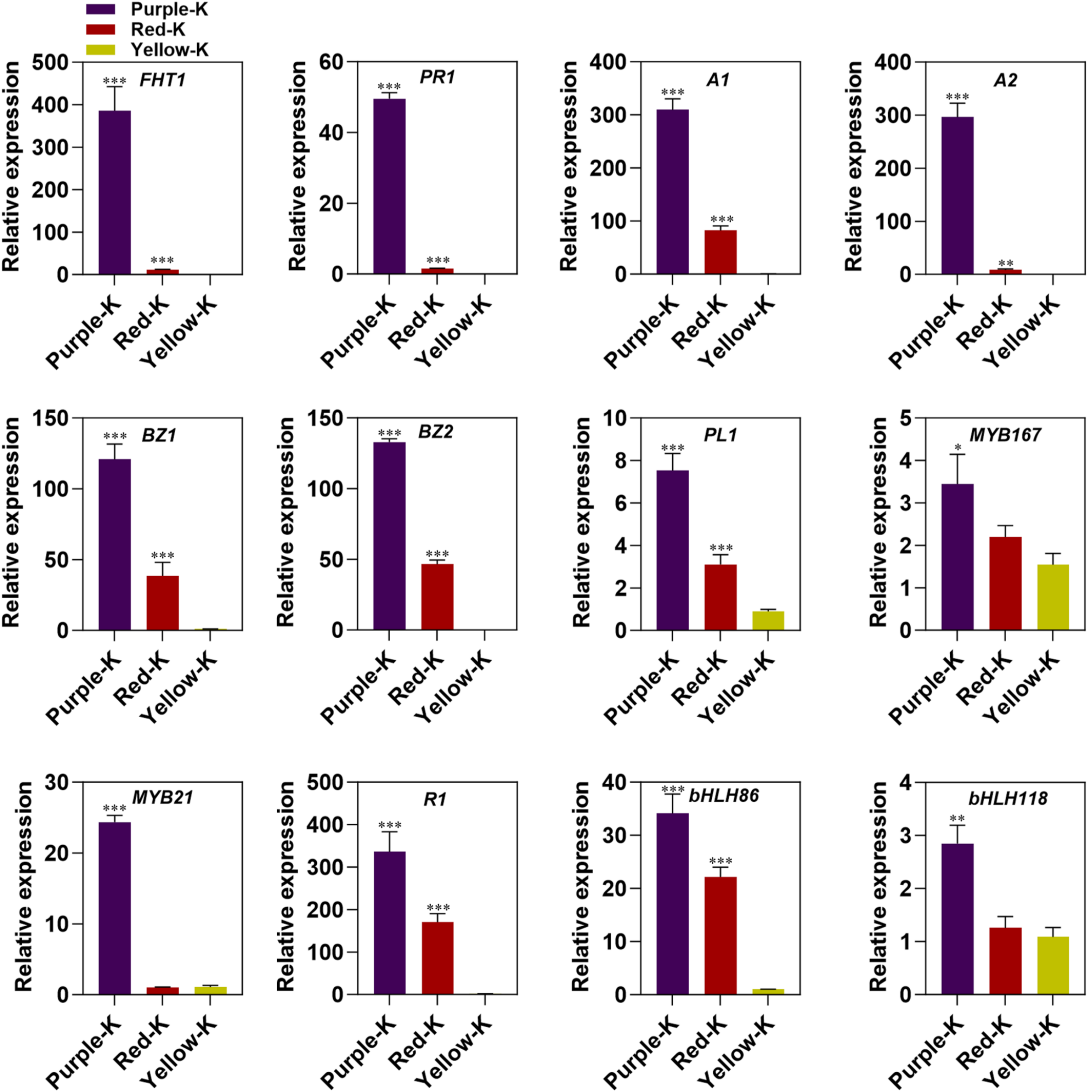
Verification of gene expression profiling using qRT-PCR. Values are means ± SD (n=3). *P < 0.05, **P < 0.01 and ***P < 0.001 indicate significant difference compared to Yellow-K.

A total of 12 DEGs, including 6 structural genes, 3 MYB genes and 3 bHLH genes were selected to verify the reliability of the results of RNA-Seq. The expression levels of these genes in three maize kernel groups were analyzed by qRT-PCR (**Figure 7**). The results showed that the expression trend of these genes was generally consistent with that of the RNA-seq results, indicating that the RNA data are valid and reliable.

## 4 Discussion

### 4.1 Anthocyanin-rich maize kernels as a source of bioactive compounds

Anthocyanin-rich maize kernels are considered a valuable source of antioxidant compounds with potential health benefits. Compared to yellow kernels, purple and red maize kernels exhibit higher anthocyanin content and enhanced biological activities, such as antioxidant, antimicrobial, and anti-obesity properties (Hua et al., 2023; Li et al., 2012; Harakotr et al., 2014a). Therefore, kernel color may serve not only as a visible agronomic trait but also as a key phenotypic marker for screening germplasm with putative nutritional quality. In maize, anthocyanins primarily accumulate in the pericarp and/or aleurone layer (Harakotr et al., 2014a; Zhao et al., 2009). In this study, we dissected this trait using three inbred lines: Yellow-K, Red-K, and Purple-K. The purple kernels accumulated pigments in both the pericarp and aleurone layer, whereas the red kernels accumulated pigments only in the pericarp (**Figure 1A**). This distinct tissue distribution immediately suggested the possibility of differential activation of layer-specific genetic programs. The coloration of maize kernels is determined by the composition and relative abundance of anthocyanins, which give rise to hues ranging from red, purple to blue (Cui et al., 2012; Nankar et al., 2016). Our integrated multi-omics analysis presented here offers insights into molecular pathway potentially linking genotype to color phenotype.

### 4.2 Divergent anthocyanin modification pathways may contribute to kernel color differences

Although both pigmented genotypes accumulate high levels of anthocyanins, they display fundamentally divergent downstream modification trajectories—a central finding of this study (**Figure 5**; **Supplementary Table 7**). These distinct modification patterns may offer clues to understanding their respective kernel colors. The metabolic profile of Purple−K was characterized by substantial accumulation of pelargonidin-based anthocyanins, particularly Pg3G, Pg3MG, and Pg3DiMalG in Purple−K. This suggests the presence of enhanced malonyltransferase (AAT) activity and possible dual malonylation capacity, which is consistent with previous reports. Purple−K also accumulates cyanidin 3-O-sambubioside, along with coumaroylated and glucuronosylated cyanidin derivatives, suggesting a relatively complete suite of secondary modifications. This chemical diversity, mixing cyanidins with modified pelargonidins, potentially further stabilized by extensive acylation, might contribute to the observed purple phenotype, though the exact relationship between specific modifications and final color requires further investigation. In contrast, Red−K almost lacks sambubiosides and malonylated pelargonidins, but accumulates pelargonidin 3-O-rutinosides and their 5-O-glucosides. This pattern suggests Red−K selectively retained rhamnosyltransferase and specific coumaroyltransferase activities, directing flux toward high-level accumulation of a limited set of red pigments, consistent with previous report of these anthocyanin modifications in colored maize germplasm (Harakotr et al., 2014b). These complementary modification profiles raise the possibility that purple and red kernels may carry distinct regulatory alleles controlling modification enzyme expression. Purple−K may retain a broad modification network associated with pigment diversity and stability, while Red−K may have specialized in a subset of activities for targeted red pigment accumulation. This divergence may potentially contribute to their distinctive kernel colors, further genetic investigation is required.

To explore the enzymes that may underlie these divergent branches, we surveyed the transcriptome for UGT and BAHD genes with expression patterns correlated with the metabolite profiles. Among UGT members, *BZ1* was more highly expressed in Purple-K, potentially enhancing Pg3G availability for acylation, whereas *Zm00001eb337060* was more abundant in Red-K, possibly reflecting distinct flavonol glycosylation patterns between the two genotypes. Among BAHD members, *AAT1* showed moderately higher expression in Purple-K than in Red-K, coinciding with substantially elevated levels of Pg3MG and Pg3DiMalG (>18-fold and >3,000-fold, respectively). Additionally, *Zm00001eb191180* was similarly upregulated in both pigmented genotypes, while *Zm00001eb095790* was upregulated in Purple-K and may be associated with p-coumaroylated derivatives (**Figure 5**; **Supplementary Table 6**). These candidates provide potential leads for further functional investigation. However, beyond gene expression, other factors such as post−transcriptional regulation, enzyme catalytic efficiency, or substrate availability, may also contribute to the observed differences. Further heterologous expression and biochemical characterization of these candidate BAHD enzymes would be necessary to determine their specific activities toward pelargonidin substrates and to explore the causal relationships.

### 4.3 Coordinated upregulation of core structural genes

The differential accumulation of metabolites is likely associated with differential gene expression regulation. Transcriptomic analysis revealed that red and purple kernels shared a core set of anthocyanin structural genes that were coordinately upregulated in both Purple−K and Red−K compared to Yellow−K, suggesting a possible shared enhancement of general flavonoid flux (**Figure 5**; **Supplementary Table 6**). Among the 47 differentially expressed structural genes, 22 core genes were coordinately upregulated in both Purple−K and Red−K relative to Yellow−K, covering the entire pathway from phenylpropanoid entry (PAL) to vacuolar sequestration (GST). However, the key to their divergence lay in the activation of two specific transcriptional programs uniquely active in purple kernels: enhanced expression of genes at key rate-limiting steps. *DFR* catalyzes the conversion of three primary substrates (dihydrokaempferol and dihydroquercetin) into colorless leucoanthocyanidins. Notably, the specific *DFR* genes (*A1*, *CNCR2* and Zm00001eb237050) showed higher expression in Purple−K, which correlated with increased leucocyanidin pools. Subsequently, ANS (also known as leucoanthocyanidin dioxygenase) catalyzes the oxidative conversion of colorless leucoanthocyanidins (e.g., leucocyanidin) into their corresponding colored anthocyanidins (e.g., cyanidin) (Jun et al., 2018). Most decisively, key *ANS* genes (notably *A2* and *ZAR8*, with fivefold and twofold higher expression in Purple-K than in Red-K, respectively) were dramatically upregulated, along with the uniquely upregulated Zm00001eb287000. As ANS catalyzes the committed step of forming colored anthocyanidins, this upregulation may be associated with to the higher accumulation of all three anthocyanidin aglycones in Purple-K.

### 4.4 Potential transcription factors associated with tissue-specific anthocyanin regulation

Transcription factors are involved in the regulation of anthocyanin biosynthesis, with the MYB and bHLH families recognized as key regulators of this pathway (Gonzalez et al., 2008). In maize, the bHLH-MYB transcriptional complex, with *R1* and *C1* as key components, has been shown to activate multiple structural genes in the anthocyanin pathway, while additional family members may contribute to fine-tuning anthocyanin accumulation in different tissues (Grotewold et al., 1994; Feller et al., 2006; Burr et al., 1996). For instance, *Purple aleurone1* (*PR1*), under the control of *C1* and *R1*, modulates pelargonidin accumulation specifically in the aleurone layer (Sharma et al., 2011). In this study, co-expression network analysis identified a key module comprising 4 MYB and 10 bHLH transcription factors (**Figure 6**; **Supplementary Table 9**), including known regulators such as *PL1* (Cone et al., 1993) and *R1* (Riaz et al., 2019), whose expression levels were positively correlated with anthocyanin content and significantly elevated in Purple-K (**Figure 4**). The pericarp-associated MYB *PL1* and bHLH *R1* were highly expressed in both pigmented lines and nearly absent in Yellow-K, with strong co-expression (r = 0.99) suggesting they may function together in regulating pericarp pigmentation. These findings are consistent with the conserved MBW (MYB-bHLH-WD40) regulatory mechanism documented in other species, such as *Arabidopsis*, where TT8 forms a complex with PAP1/MYB75 and TTG1 to activate anthocyanin structural genes (Jun et al., 2018).

Interestingly, the classical aleurone-specific MYB regulator *C1* (Zm00001eb373660) and the characterized bHLH regulator *B1* (*Booster*; Zm00001eb074320) were not included in the GEturquoise module and showed low correlation with anthocyanin structural genes (**Supplementary Table 9**). *C1* expression was detected exclusively in Red-K, with no expression in Purple-K or Yellow-K, while *B1* showed negligible expression in all three lines. These observations suggest the possibility that in the germplasms examined here, anthocyanin regulation may be influenced by alternative MYB-bHLH complexes, with *R1* potentially serving as the predominant bHLH partner rather than *B1*. This tissue-specific partitioning of regulatory complexes has been previously documented in maize, where different combinations of MYB and bHLH factors control pigmentation in distinct tissues (Cone et al., 1993; Burr et al., 1996). Three MYB genes, *MYB21*, *MYB158*, and *MYB56*, and one bHLH gene, *bHLH118*, showed preferential expression in Purple-K, with reduced expression in Red-K and Yellow-K (**Figure 6A**). These candidates exhibited co-expression with *PR1* and *A2*, two structural genes highly expressed in Purple-K, raising the possibility that they may participate in an alternative regulatory complex associated with aleurone pigmentation. In Red-K, an uncharacterized MYB gene (Zm00001eb234690) showed strong Red-K-enriched expression and co-expression with *C1* (r = 0.96) (**Supplementary Table 9**). The bHLH gene *bHLH82* exhibited exceptionally high expression in Red-K and co-expressed with the Red-K-enriched chalcone synthase gene *CHLS8*, making it a candidate gene for further investigation of its possible role in pericarp pigmentation. The candidate transcription factors identified in this study will require functional validation in future work. The presence of conserved DNA-binding domains (**Supplementary Figures 2**, **3**) and cognate cis-motifs in target promoters (**Supplementary Figure 4**) may be indirectly suggestive of their potential regulatory roles. Nevertheless, direct biochemical experiments, such as dual−luciferase reporter assays to examine whether these TFs might activate the key structural genes *A1* or *A2* promoters, along with genetic approaches, would be valuable to help elucidate the mechanisms underlying the divergent anthocyanin modification pathways in Purple−K and Red−K.

### 4.5 Hormonal regulation may integrate with the transcriptional network

Plant hormones, particularly jasmonic acid (JA), is also involved in the regulation of anthocyanin biosynthesis (Shi et al., 2023). The JA signaling pathway is negatively regulated by JAZ proteins, which function as transcriptional repressors of JA-responsive transcription factors (An et al., 2021; Zhang et al., 2015). Upon JA perception, JAZ proteins are degraded, leading to the release of MYB–bHLH complexes and the subsequent activation of anthocyanin biosynthesis. In this study, JAZ repressor genes showed differential expression among the three kernel types (**Figure 6B**; **Supplementary Table 10**). *ZmJAZ18* and *ZmJAZ13* were downregulated in Purple-K compared to Yellow-K, consistent with JA-mediated derepression of anthocyanin biosynthesis. The result was consistent with previous report of MdJAZ18 as a negative regulator of JA-mediated anthocyanin biosynthesis (Liu et al., 2017). In contrast, *ZmJAZ22*, *ZmJAZ11*, *ZmJAZ21*, and *ZmJAZ29* were upregulated in Red-K, suggesting functional diversification among JAZ family members in maize. Their lower expression in Purple-K is consistent with the elevated anthocyanin content observed in this genotype. Several bHLH transcription factors targeted by JAZ repression were markedly upregulated in pigmented kernels. *R1* and *in1* showed higher expression in Purple-K and Red-K than in Yellow-K. While *bHLH137* and *bHLH147* were higher expression only in Purple-K (**Figure 6B**, **Supplementary Table 11**). The reported functional similarity between maize *R1* and *Arabidopsis TT8* (Baudry et al., 2006) may indicate the evolutionary conservation of this regulatory node between monocots and dicots.

However, we recognize that the differential expression of *JAZs* may alternatively arise from background allelic variation among the three landrace genotypes. Investigations using controlled genetic backgrounds, such as recombinant inbred lines or gene-edited mutants, would be valuable to further dissect the relationship between JA signaling and the divergent anthocyanin modification pathways. Future work should focus on characterizing the potential interactions within the identified TF module and exploring how plant hormones might integrate with this genetic framework to modulate pigment accumulation in maize kernels. Emerging single-cell transcriptomics and spatial metabolomics will further elucidate the tissue-specific regulatory dynamics of anthocyanin biosynthesis (Zhang et al., 2025a).

## 5 Conclusions

This study provides a comprehensive multi-omics analysis of anthocyanin accumulation and its potential regulatory network in maize kernels with distinct coloration. By integrating phenotypic, metabolomic, transcriptomic, and transcriptional regulatory data, we elucidated the metabolic and molecular basis underlying color formation in purple and red maize kernels. (1) tissue-specific pigmentation—both pericarp and aleurone layers in purple kernels, versus pericarp only in red; (2) Purple−K and Red−K share upregulated core structural genes but diverge completely in decorative modifications: Purple−K specializes in malonylation and sambubiosylation, massively accumulating Pg3DiMalG; Red−K specializes in rutinosylation and 5−O−glycosylation; (3) coordinated upregulation of structural genes (*PALs, F3H, A1, A2, BZ1, BZ2*) correlating with anthocyanin accumulation, and exclusively upregulated structural genes (*PR1 and A2*) were in Purple−K; (4) identification of key bHLH (e.g., *R1*) and MYB (e.g., *PL1*) transcription factors; and (5) JA-mediated regulation is suggested by *ZmJAZ* repression and subsequent bHLH activation. These results provide molecular targets for breeding anthocyanin-rich maize and highlight the need to further study epigenetic and hormone crosstalk mechanisms.

## Data Availability

The datasets presented in this study can be found in online repositories. The names of the repository/repositories and accession number(s) can be found in the article/**Supplementary Material**.

## Supplementary material

Supplementary Figure 1 Differentially expressed genes in three maize kernels. **(A)** Volcano plot of DEGs. Red and blue dots represent up- and downregulated metabolites, respectively. **(B)** K-means DEG clustering algorithm.

Supplementary Figure 2 Alignment of the amino acid sequence of candidate MYBs with homolog from *Arabidopsis*. Red rectangles, conserved R2R3-MYB domain; blue triangles, bHLH interaction domain.

Supplementary Figure 3 Alignment of the amino acid sequence of candidate bHLHs with homolog from *Arabidopsis*. Orange rectangles, conserved bHLH domain.

Supplementary Figure 4 Promoter analysis of *A1* and *A2* for MYB- and bHLH-binding motifs. Green rectangles indicate MYB-recognizing elements (MRE, ANCNNNC); yellow rectangles indicate bHLH-recognizing element (BRE, CANNTG or CACN(A/C/T)(G/T)).
