# Supplementary figures and images for: Integrated transcriptomic and metabolomic profiles analysis reveals a potential gene−metabolite network associated with anthocyanin−mediated color variation in maize kernels
Source: Front Plant Sci. 2026 May 13;17:1828668. doi: 10.3389/fpls.2026.1828668 (PMC13212506; doi:10.3389/fpls.2026.1828668)

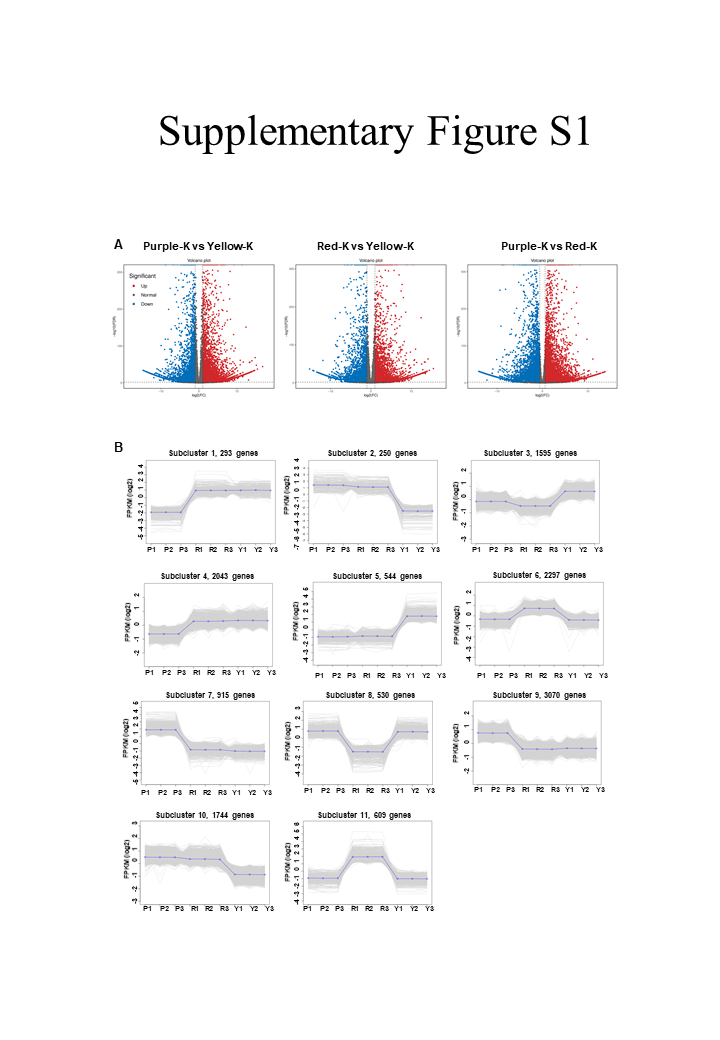

Supplement: Supplementary Figure 1 — Differentially expressed genes in three maize kernels. (A) Volcano plot of DEGs. Red and blue dots represent up- and downregulated metabolites, respectively. (B) K-means DEG clustering algorithm. [file Image1.tif]

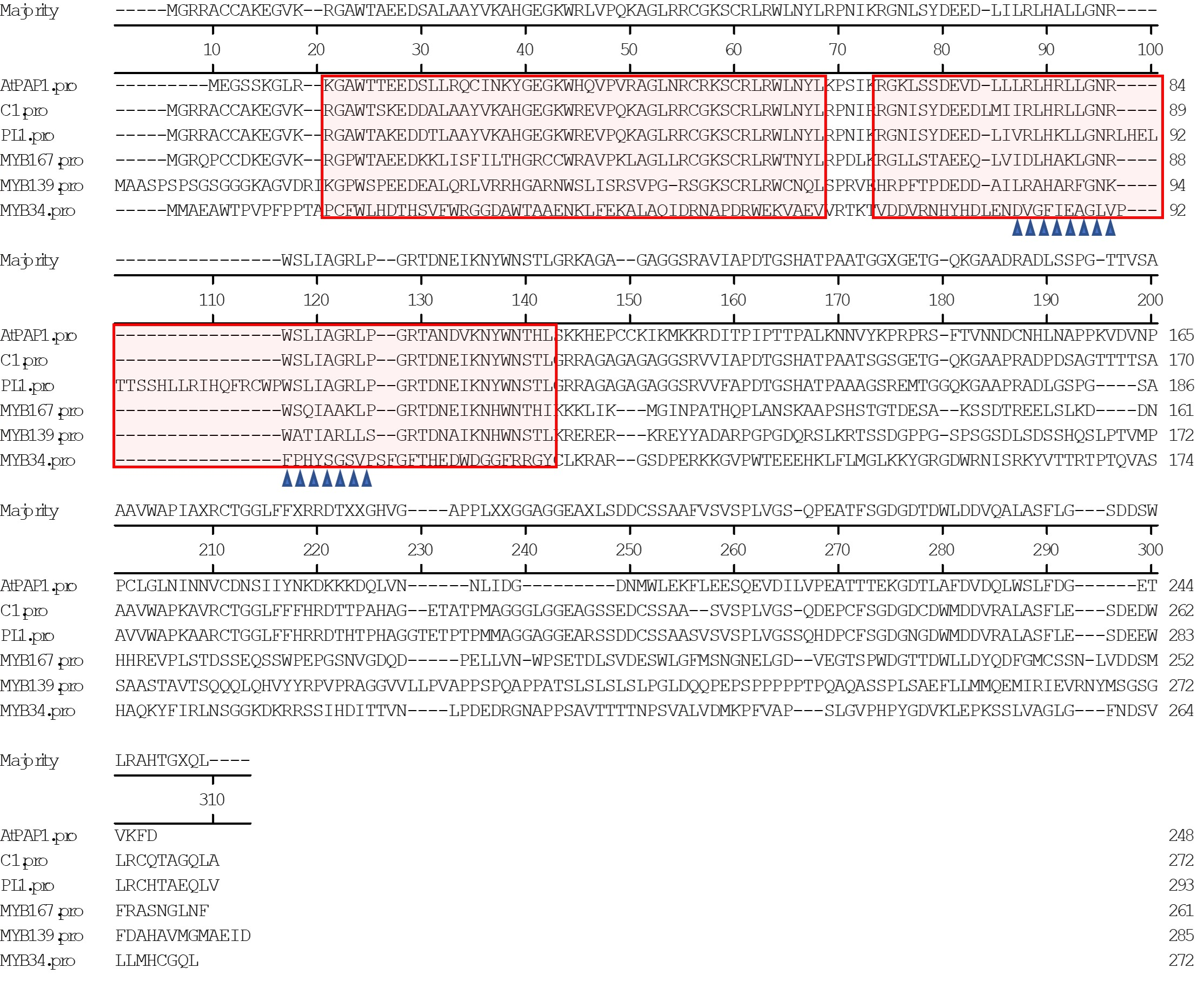

Supplement: Supplementary Figure 2 — Alignment of the amino acid sequence of candidate MYBs with homolog from Arabidopsis. Red rectangles, conserved R2R3-MYB domain; blue triangles, bHLH interaction domain. [file Image2.tif]

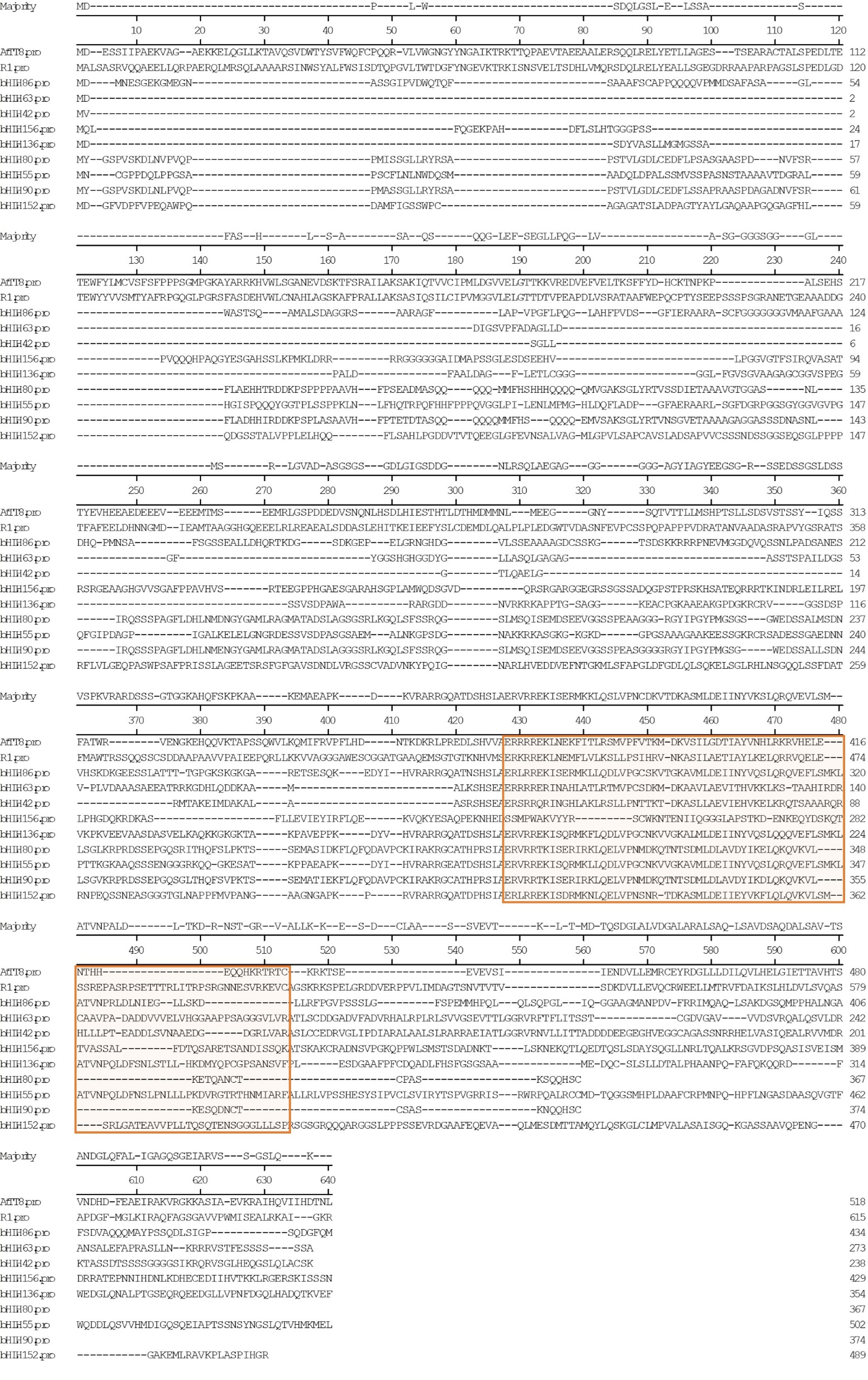

Supplement: Supplementary Figure 3 — Alignment of the amino acid sequence of candidate bHLHs with homolog from Arabidopsis. Orange rectangles, conserved bHLH domain. [file Image3.tif]

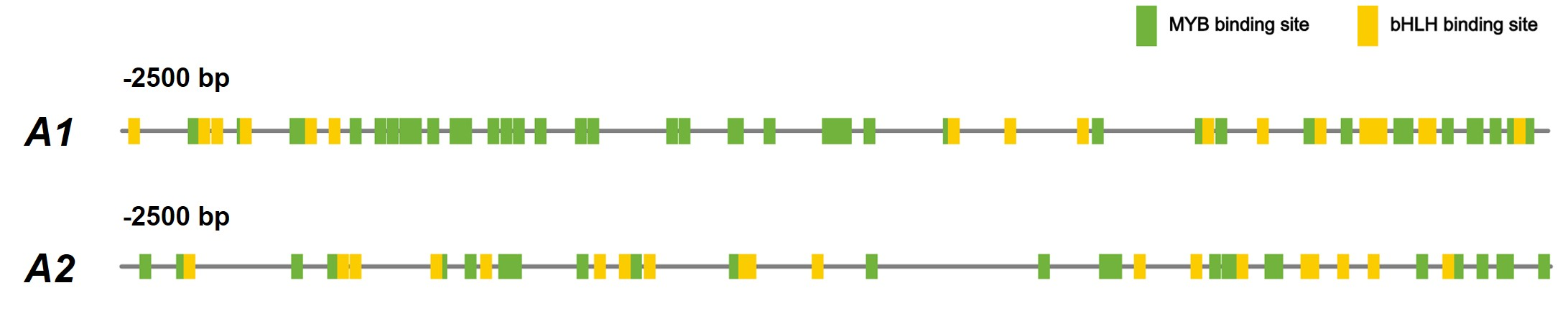

Supplement: Supplementary Figure 4 — Promoter analysis of A1 and A2 for MYB- and bHLH-binding motifs. Green rectangles indicate MYB-recognizing elements (MRE, ANCNNNC); yellow rectangles indicate bHLH-recognizing element (BRE, CANNTG or CACN(A/C/T)(G/T)). [file Image4.tif]
